# Primary Synovial Sarcoma (SS) of the digestive system: a molecular and clinicopathological study of fifteen cases

**DOI:** 10.1186/s13569-015-0021-3

**Published:** 2015-02-12

**Authors:** Salvatore Romeo, Sabrina Rossi, Marthelena Acosta Marín, Fabio Canal, Marta Sbaraglia, Licia Laurino, Guido Mazzoleni, Maria Cristina Montesco, Laura Valori, Marta Campo Dell’Orto, Andrea Gianatti, Alexander Joseph Lazar, Angelo Paolo Dei Tos

**Affiliations:** Department of Pathology, Treviso Regional Hospital, Piazza Ospedale 1, 31100 Treviso, Italy; Departments of Pathology, The University of Texas M. D. Anderson Cancer Center, Houston, TX USA; Department of Pathology, Bolzano Regional Hospital, Bolzano, Italy; Department of Pathology, Padua University Medical School, Padua, Italy; Department of Pathology, Ospedali Riuniti di Bergamo, Bergamo, Italy

**Keywords:** Synovial sarcomas, Pathology, Differential diagnosis, Digestive tract, Sarcoma

## Abstract

**Background:**

Recently a few cases of synovial sarcoma (SS) of the abdominal viscera have been reported, raising awareness about the potential for confusion between this entity and KIT-negative gastrointestinal stromal tumors (GIST). We report the clinicopathological, immunophenotypical and molecular features of fifteen more SS occurring in the stomach (8 cases), epigastric region (one case), small intestine (one case), large intestine (three cases), involving both the terminal ileum and the caecum (one case) and liver (one case).

**Methods:**

Immunostains for SMA, DESMIN, CD34, CD117, S100, EMA, CK AE1/3, TLE1, CD56, CD99, BCL2, DOG1 were performed. Rearrangement of *SS18* gene region was screened in all cases: by conventional karyotype in one case, the remaining cases were screened either by interphase FISH or Q-PCR or both.

**Results:**

Ten patients were male and five female, with an age range of 17–61 years (median 44). Tumor size ranged from 2 to 15 cm (median 8). Mitoses per 10 HPF ranged from 4 to 27 (median 9.5). Eleven tumors were monophasic fibrous SS, one biphasic SS and three poorly differentiated SS. SMA, Desmin, CD34, CD117 and S100 were negative in all cases, whereas EMA and/or CK AE1/AE3 were positive in all cases. TLE1, BCL2 and CD56 were positive in all tested cases. DOG1 was positive in one case. *SS18* gene region rearrangement was demonstrated in all cases. A fusion transcript was amplified in eight cases: either SS18-SSX2 or SS18-SSX1 respectively in four cases each.

**Conclusions:**

SS is increasingly recognized at visceral sites. Molecular analyses play a key role when dealing with usual histotypes in unusual sites. Correct diagnosis is crucial for appropriate therapy.

## Introduction

Synovial sarcoma (SS) is a mesenchymal malignant tumour that accounts for approximately 10% of all soft tissue sarcomas [1]. It usually occurs in the lower limbs of children and young adults, with the knee region being the most frequently affected area [1]. Three main histological variants of SS have been recognized: the monophasic, biphasic and poorly differentiated subtypes [1]. Both the monophasic and biphasic variants feature a spindle cell population set in a variable collagenous background with a hemangiopericytoma (HPC)-like vascular pattern [1]. An epithelial component is present in the biphasic variant, with solid nests and glandular or tubular structures [1]. The existence of a predominantly monophasic epithelial pattern has been reported, too [2]. In approximately 20% of cases, SS exhibits undifferentiated, high-grade morphology and is usually indicated as “poorly differentiated” SS (PDSS) [3]. Three main groups of PDSS can be identified: one exhibiting round cell morphology associated with necrosis and high mitotic count; another characterized by the presence of larger cells, with polygonal cytoplasm which may feature rhabdoid morphology; and a third group presenting as high-grade spindle cell tumors often featuring a “herringbone” growth pattern [3,4].

SS subtypes share a common genetic alteration: a translocation involving chromosomes X and 18. This translocation results in three alternative fusion products of the *SS18* gene (previously known as *SYT*) (on chromosome 18) with either *SSX1*, or *SSX2 or SSX4* gene (on chromosome X) [1]. This knowledge provides useful ancillary diagnostic tools [1] for identification of the specific translocation by interphase FISH analysis, with probes flanking the breakpoints, and amplification of the specific chimeric transcript by RT-PCR techniques [5].

SS rarely occurs in unusual sites including: the head and neck region [6,7], mediastinum [8], larynx and hypopharynx [9], nerves [10], blood vessels [11,12], heart [13], abdominal cavity [14], gastrointestinal tract [15-23] and liver [24,25]. In routine activity it may be difficult to distinguish SS occurring in the digestive tract from other mesenchymal neoplasms, mainly GIST (gastrointestinal stromal tumour). However, this distinction is crucial to ensure a correct therapeutic approach. Here we report the clinicopathological, immunohistochemical and molecular genetic data of fifteen cases of SS occurring in the digestive system. We aim to improve knowledge on this entity and stress the importance of correct differential diagnosis for appropriate therapeutic management.

## Materials and methods

### Patients

Fifteen cases of SS of the abdominal viscera were collected from three Italian institutions, the consultation files of one of the Authors (APDT) and Departments of Pathology, The University of Texas M. D. Anderson Cancer Center, Houston, TX, USA. Patients’ clinical records were retrieved. Follow-up information was available for 11 patients (Table 1). All samples were handled in a coded fashion, and all procedures were performed according to the ethical guidelines of the local institutions.

**Table 1:** **Clinicopathologic features of 14 synovial sarcomas of the digestive system**

**Case #**	**Site**	**Size**	**Gender**	**Age**	**Follow up**	**Metastases**
1	Gastric body	8	F	50	Lost	
2	Cardias	6	M	36	AWD@36	Liver
3	Gastric	2	M	37	Recent	
4	Gastric	NR	M	26	AWD@185	Liver, Lungs
5	Gastric	10	M	58	DOD@6	
6	Gastric	10	M	21	Lost@48	
7	Gastric	6	M	36	Lost@12	
8	Gastric	3.8	F	54	Recent	
9	Epigastrium	13	F	57	AWD@7	
10	Ileum	8	M	49	DOD@60	
11	Large bowel	5.5	M	40	NED@132	
12	Rectosigmoid colon	6.3	F	44	DOD@47	
13	Rectosigmoid colon	6.3	F	44	DOD@47	
14	Ileum/Colon	7.5	M	17	NED@108	
15	Liver	15	M	61	AWD@12	

Legend: Size is given in centimeter; NR: not reported, Follow Up is in months; DOD: dead of disease; NED: not evidence of disease; AWD: alive with disease; Lost: lost to follow up.

### Pathology assessment and immunohistochemistry

All the cases were reviewed for diagnostic confirmation and both necrosis extent and mitoses count evaluated, grading provided according to French National Federation of Cancer Centers (FNLCC) (Table 2). In a subset of cases neoadjuvant chemotherapy was applied and pre-chemo biopsies were not available (Table 2); the values for mitoses and necrosis are no longer relevant for these cases as pre-treated specimens cannot be accurately graded under the FNCLCC system.

**Table 2:** **Morphological features of 15 synovial sarcomas of the digestive system**

**Case #**	**Type**	**Mitoses**	**Necrosis**	**Grading**	**Involvement of**		
					**Perivisceral soft tissue**	**Peritoneum**	**Adjacent organ**
1	M	7	1	2	None	None	None
2	PD	11	1	2	Adventitial tissue	Yes	None
3	M	6	0	2	None	None	None
4	M	P	P	P	Adventitial tissue	Yes	Pancreas
5	M	12	1	2	Adventitial tissue	Yes	Pancreas
6	M	P	P	P	None	None	None
7	B	27	1	3	None	None	None
8	M	14	1	2	None	None	None
9	M	8	2	3	Stomach, duodenum and liver	Yes	None
10	M	5	1	2	None	None	None
11	M	13	1	2	Perivisceral adipose tissue	None	None
12	PD	P	P	P	Perirectal adipose tissue	None	None
13	PD	P	P	P	None	None	None
14	M	4	1	2	None	None	None
15	M	P	P	P	None	None	None

Legend: M: monophasic, B: biphasic, PD: poorly differentiated, mitoses are per 10 HPF, P: pretreated. Necrosis is reported as: 0 for no necrosis, 1 for <50% tumor necrosis, 2 for ≥ 50% tumor necrosis.

Immunostaining was performed for EMA, cytokeratin AE1/AE3, SMA, Desmin, CD34, CD117, S-100, CD99, CD56 and TLE1. Four-μm sections of formalin-fixed paraffin-embedded material were used according to standard laboratory procedures. Details of the antibodies used are given in Table 3.

**Table 3:** **Details of the antibodies used in this study**

**Antibody**	**Clone**	**Producer**	**Diluition**	**Antigen retrieval**
EMA	E29	Dako, Glostrup, Denmark	Prediluted	Flex (Dako)
CKAE1/AE3	Polyclonal	Dako, Glostrup, Denmark	Prediluted	Flex (Dako)
SMA	1A4	Dako, Glostrup, Denmark	Prediluted	Flex (Dako)
DESM	D33	Dako, Glostrup, Denmark	Prediluted	Flex (Dako)
CD34	QBend-10	Dako, Glostrup, Denmark	prediluited	Flex (Dako)
CD117	Polyclonal	Dako, Glostrup, Denmark	1/700	Flex (Dako)
S-100	Polyclonal	Dako, Glostrup, Denmark	Prediluted	Flex (Dako)
TLE1	c-9121	Santa Cruz Biochemicals, Santa Cruz, CA, USA	1:100	Flex (Dako)
BCL2	124	Dako, Glostrup, Denmark	Prediluted	Flex (Dako)
CD56	123C3	Dako, Glostrup, Denmark	Prediluted	Flex (Dako)
CD99	MIC2	Dako, Glostrup, Denmark	Prediluted	Flex (Dako)
DOG1	K9	Novocastra, NewCastle, UK	1:100	Flex (Dako)

### Conventional karyotype

For case 14 conventional karyotype was performed. Cell culture, harvest conditions, and karyotyping were performed according to standard protocols.

### Interphase FISH

Fluorescent *in situ* hybridisation (FISH) was performed on 5 μm paraffin-embedded tissue sections using the LSI SYT (18q11.2) Dual Color Break Apart Rearrangement Probe set (Vysis, Downers Grove, IL, USA).

Hybridisation was performed according to the manufacturer’s protocol. Slides were mounted and counterstained with anti-fade DAPI (Vysis, Downers Grove, IL, USA), visualized using an epifluorescent microscope (Olympus BX61) and analysed with FISH analysis software (Genetix-Cytovision 4.5.1). 300 interphase nuclei were analyzed.

### Q-PCR

Ten to fifteen 15 μm-thick sections from paraffin-embedded tissue were de-paraffinized twice using xylene, washed twice with absolute ethanol followed by TNE1X, resuspended in 250 μl of ATL buffer (Qiagen) with the addition of proteinase K (Qiagen), and incubated for 72 hours at 55°C under moderate shaking. The percentage of tumor cells, as calculated from the HE-stained slides, was at least 70%. Subsequently, RNA was extracted with TRIzol-LS Reagent (Gibco BRL), according to the manufacturer’s instructions. RNA pellets were resuspended in 10–20 μl of RNAse-free water and stored at −80°C. 5 μg of total RNA were reverse transcribed in a total volume of 20 μl using specific reverse primers for *SSX* and *BETA2M* genes, respectively. Samples were incubated at 42°C for 1 hour, then at 72°C for 15 minutes. PCR amplification of each sample and a 1:20 dilution were performed in duplicate using 96-well plates in 25 μl reaction mixture containing 300 nM of each primer, 200 nM of each probe (SSX1-SSX2) or 100 nM probe BETA2M and 2X TaqMan Gene Expression Master Mix (Applied Biosystems, CA), on Applied Biosystems StepOnePlus Real-Time PCR Systems (Applied Byosystem). (For primer and probe sequences, see Table 4). Thermal cycling conditions were 2 minutes at 50°C, 10 minutes at 95°C, then 50 cycles for three PCR steps consisting of 30 seconds at 95°C and 1 minute at 60°C. Eight cases were studied by Q-PCR, for all of them a positive control product was amplified.

**Table 4:** **Sequences of the primers and probes used in this study**

**PRIMER/PROBE name**	**Sequence**
SS18 (forward)	AGAGGCCTTATGGATATGACCAGAT
SSXC (reverse)	CRTTTTGTGGGCCAGATGC
BETA2M+ (forward)	TGACTTTGTCACAGCCCAAGATA
BETA2M- (reverse)	AATCCAAATGCGGCATCTTC
SSX1 probe	TCCCTTCGAATCATTTTCGTCCTCTGCT
SSX2 probe	TCTGGCACTTCCTCCGAATCATTTCCTT
BETA2M probe	TGATGCTGCTTACATGTCTCGATCCCA

## Results

In the identified fourteen cases of digestive system SS a male prevalence was found: male/female ratio was 3:1. Age at clinical presentation ranged from 17 to 61 years (median 44 years) (Table 1). Size ranged from 2 cm to 15 cm (median 8 cm) (Table 1). Eight cases were occurring in the stomach, one case in the epigastric region, one case in the ileum, three cases in the large intestine, one case was involving both the terminal ileum and the caecum and one case in the liver (Table 1).

**Figure 1:**
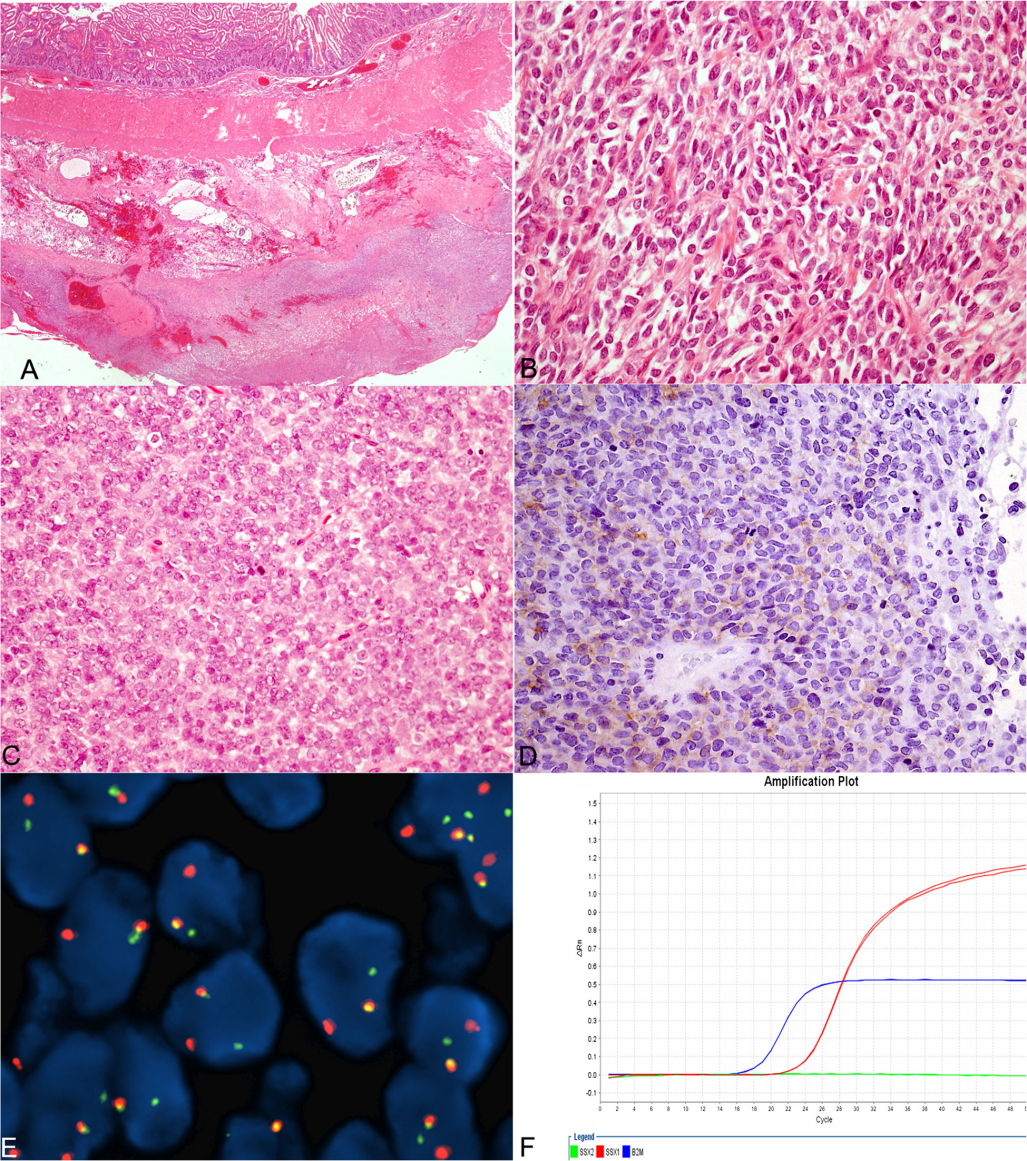
**Digestive tract SS features: A- Neoplastic cells were involving gastric wall with focal extension to nearby perivisceral soft tissue and serosal ulceration (case 2, 12.5 X original magnification, HE staining).** **B**- Most of the cases were composed of monomorphic spindle cells (case 9, 400 X original magnification, HE staining). **C**- Poorly differentiated SS featuring monomorphic round cells with elevated mitotic rate were encountered in 3 cases (case 4, 400 X original magnification, HE staining). **D**- Focal positivity for DOG1 staining was found in one case(Case 9, 400 X original magnification). **E**- Interphase FISH showed split a part of the two signal in most of the nuclei (case 9, original 1000X magnification). **F**- A fusion transcript SS18-SSX1 was amplified by Q-PCR (plot showing the amplification curves of SS18-SSX1, SS18-SSX2 and beta-microglobulin).

Five of the eight tumors arising in the stomach tract were confined to the wall (without serosal involvement); one extended into the perivisceral soft tissue, peritoneum and omentum (Figure 1A) and two involved the pancreas (Table 2). The tumor located in the epigastric region was adherent to the stomach and duodenal wall and focally attached to the liver (Table 2). Three tumors were involving the large bowel with extension in the perivisceral adipose tissue in two of them. The case affecting the ileum was limited to the ileum wall, similarly also the case involving both ileum and caecum was limited to the visceral wall (Table 2). The one in the liver was confined to the parenchyma without ulceration of the Glisson’s capsule (Table 2).

Microscopically, eleven of the tumours consisted of monotonous spindle cell proliferation, with scant intercellular eosinophilic collagenous stroma (Figure 1B) (Table 2). A focal HPC-like vascular network was observed. Three cases showed poorly differentiated features (Figure 1C) and one showed biphasic features with both spindle cells components and tubules formation (Table 2).

Mitotic count ranged from 4 to 27 mitoses/10 HPF (median 9.5) (Table 2). Immunoreactivity for EMA was found in all tested cases ranging from focal to strong and diffuse (Figure 1D) (Table 5). Focal positivity for immunostains for cytokeratin AE1-AE3 was found in 8 cases (Table 5). All tested cases were positive for TLE1, BCL2 and CD99 (Table 5). No expression of CD117, SMA, DESMIN, CD34 and S-100 protein was found. DOG1 was focally expressed in one case (case 9, Table 5)

**Table 5:** **Results of the performed immunostains and fusion type in 15 synovial sarcomas of the digestive system**

**Case #**	**EMA**	**CKAE1/AE3**	**SMA**	**DESMIN**	**CD34**	**CD117**	**S-100**	**BCL2**	**CD99**	**CD56**	**DOG1**	**TLE1**	**Fusion type**
1	+	-	-	-	-	-	-	+	+	+	-	+	SS18-SSX1
2	+	-	-	-	-	-	-	+	+	+	-	+	N/A
3	+	+	-	-	-	-	-	+	+	+	-	+	N/A
4	+	-	NP	NP	NP	-	-	NP	NP	NP	NP	NP	N/A
5	+	-	NP	-	-	-	-	NP	NP	NP	NP	NP	SS18-SSX1
6	+	-	NP	NP	NP	-	-	NP	NP	NP	NP	NP	N/A
7	NP	+	NP	NP	-	-	NP	NP	NP	NP	NP	NP	SS18-SSX2
8	+	+	-	-	-	-	-	+	+	+	-	+	SS18-SSX1
9	+	+	-	-	-	-	-	+	+	+	+	+	N/A
10	+	+	-	-	-	-	-	+	+	+	-	+	SS18-SSX2
11	+	-	-	-	-	-	-	+	+	+	-	+	SS18-SSX2
12	+	+	NP	NP	NP	-	-	+	+	NP	NP	NP	SS18-SSX2
13	+	+	NP	NP	NP	-	-	+	+	NP	NP	NP	SS18-SSX1
14	+	-	NP	-	-	-	-	+	+	NP	NP	NP	N/A
15	+	+	-	-	-	-	-	+	+	+	-	+	N/A

.

All cases showed *SS18* gene region rearrangement. Case 14 showed 46, XY, t(X; 18)(p11;q11) karyotype (data not shown). The remaining cases were assessed either by interphase FISH (Figure 1E) or by RT-PCR or both. Eight cases were analyzed by RT-PCR: either SS18-SSX2 fusion transcript or SS18-SSX1 fusion transcript was identified in 4 cases each (Figure 1F, Table 5).

Follow up was available for 11 patients: range from 6 to 185 months (47 median) (Table 1).

Based on clinical, morphological, immunophenotypical and molecular data a diagnosis of primary SS of the digestive tract was formulated: monophasic synovial sarcoma in eleven cases, biphasic SS in one case and poorly differentiated SS in three cases.

## Discussion

SS is characterized by a complex, relatively distinctive immunophenotype, which includes co-expression of mesenchymal (vimentin) and epithelial markers (cytokeratins and EMA). Since morphological features of epithelial differentiation may be very subtle, immunostains are a valuable diagnostic aid. Cytokeratins tend to decorate most biphasic synovial sarcomas, but when dealing with the monophasic subtype, the percentage of immunopositivity falls to 60%-70%. Interestingly, cytokeratin immunoreactivity has been demonstrated only in 50% of PDSS [3] and high molecular weight cytokeratins proved to be more sensitive than low molecular weight cytokeratins. The most sensitive marker of epithelial differentiation is EMA, which stains most cases of PDSS, including those that fail to express cytokeratins [3]. Between 30% and 60% of SS express S-100 protein leading to potential confusion when dealing with the differential diagnosis between monophasic spindle-cell SS and MPNST [26,27]. To avoid diagnostic pitfalls caused by the use of single antibody, it is therefore strongly recommended to perform a panel of immunohistochemical markers.

The diagnosis of biphasic synovial sarcoma is usually straightforward, even for cases occurring in the digestive system. Regarding monophasic SS in the digestive system, the main differential diagnosis is with GIST: any mesenchymal lesion arising in the GI tract would naturally be suggestive of a diagnosis of GIST and CD117 negativity “per se” does not rule out such a possibility [28]. GISTs are usually composed of short spindled and/or epithelioid cells with perinuclear vacuolization and nuclear palisading. Recognition of the histological features, and the combination of CD117 with DOG1 staining is sufficient in the majority of cases to confirm the diagnosis of GIST [29]. However caution should be used in interpreting the results of immunohistochemistry since synovial sarcoma of the digestive system may show focal positivity for DOG 1 [30], as also exemplified in our case series. Remarkably sporadic GIST cases have been reported to be positive for cytokeratin [31,32], however EMA positivity is exceptional in GIST [23]. Leiomyosarcomas and malignant spindle cell melanomas are considered in the differential diagnosis with monophasic SS of the digestive system. However they are characterized by a higher pleomorphism and stronger immunostaining for smooth muscle markers and melanocytic markers, respectively, can usually confirm the diagnoses.

Sarcomatoid carcinoma may also be considered in the differential diagnosis, however it often exhibits conspicuous pleomorphism, stronger expression of epithelial markers, and area of conventional carcinoma are often associated with the sarcomatoid component.

Gastrointestinal clear cell sarcoma may be very difficult to distinguish form digestive system SS [33,34], also because, as previously mentioned, SS may be positive for S100 staining [26,27]. However gastrointestinal clear cell sarcomas are usually negative for epithelial markers and show rearrangement of the *EWSR1* gene [33,34]. Remarkably clear cells sarcomas occurring in the gastrointestinal tract differ from clear cell sarcomas of the soft tissue. In fact they display scattered osteoclast-type giant cells and only partial melanocytic differentiation, being debated to be a separate entity. For this reason *Stockman et al.* proposed to call it malignant gastrointestinal neuroectodermal tumor [33].

PD SS may resemble other small round cell tumors including Ewing Sarcoma/PNET, neuroblastoma, rhabdomyosarcoma and lymphoma [1]. Remarkably CD99 antigen is found also in SS [35] and epithelial markers may be absent in PDSS and focally present in Ewing Sarcoma/PNET [1]. In this setting, demonstration of rearrangement of *SSX18* or *EWSR1* is crucial for differentiating respectively PDSS from Ewing Sarcoma/PNET [1]. Also it should be remembered that CD99 positivity in synovial sarcomas does not feature the typical crisp membrane staining most often observed in Ewing’s sarcoma.

Cytogenetically, all SS variants are characterized by the reciprocal translocation t(X;18)(p11.2;q11.2), which leads, at molecular level, to the fusion between the synovial sarcoma translocation gene on chromosome 18 (*SS18*) and one of the synovial sarcoma X breakpoint (SSX) genes on chromosome X: SSX1, SSX2 and rarely with SSX4 [36-38]. Interestingly, the SS18-SSX1 translocation seems to be associated with the biphasic type [39]. The SS18 gene is unrelated to any other known gene but contains a glutamine-proline-glycine-rich region, suggestive of a transcriptional activation domain. The SSX1, SSX2 and SSX4 genes are also unrelated to other known genes and encode proteins that show a remarkable homology.

Despite initial attempt to correlate fusion type with a significantly longer disease-free survival [39,40] morphological grading is still the most important prognostic indicator [41]. Furthermore, tumor size (>5 cm), presence of neural infiltration and vascular invasion, p53 overexpression, and high Ki67 proliferation index identify subsets of SS patients with increased risk of tumor relapse [42-44].

## Conclusions

Recognizing SS is of paramount importance to ensure the right therapy, especially because SS is known to respond to ifosfamide-based systemic treatments [45]. In the clinical setting of SS of the digestive system the use of ancillary molecular techniques improves the diagnostic accuracy.
